# The Interplay of Goalkeepers and Penalty Takers Affects Their Chances of Success

**DOI:** 10.3389/fpsyg.2021.645312

**Published:** 2021-03-09

**Authors:** Benjamin Noël, John van der Kamp, Stefanie Klatt

**Affiliations:** ^1^Department of Cognitive and Team/Racket Sport Research, Institute of Exercise Training and Sport Informatics, German Sport University Cologne, Cologne, Germany; ^2^Faculty of Behavioural and Movement Sciences, Amsterdam Movement Sciences and Institute Brain and Behaviour Amsterdam, Vrije Universiteit Amsterdam, Amsterdam, Netherlands; ^3^Institute of Sports Science, University of Rostock, Rostock, Germany

**Keywords:** timing, decision making, scoring probabilities, penalty kick strategy, penalty kicking

## Abstract

Research in penalty kicking has primarily focused on spatial decision making, while temporal decision making has largely been neglected, even though it is as critical for success. Temporal decision making concerns goalkeepers choosing when to initiate their jump to the ball during the penalty taker's run-up (i.e., jump early or wait long), and penalty takers deciding where to kick the ball, either prior to the run-up or after the goalkeeper has committed to one side. We analyzed penalty takers' and goalkeepers' behavior during penalty shoot-outs at FIFA World Cups and UEFA European Championships to scrutinize if temporal aspects of decision making have an impact on penalty kick success. Results indicate that the likelihood of a penalty kick being scored depends on the combination of penalty takers' and goalkeepers' temporal decision-making strategies. Hence, moving early more often seems fruitful for goalkeepers, while penalty takers should consider varying penalty kick strategy between attempts.

## Introduction

There is plenty of research on penalty kicking in soccer focusing on either the goalkeeper or the penalty taker. However, the interplay between the two players has received much less attention. An important impetus for the research seems that the statistics (approximate success rate 75%; Kropp and Trapp, 1999; Hughes and Wells, 2002; Morya et al., 2005) indicate that penalty takers convert fewer kicks than expected and, conversely, that goalkeepers still save a considerable number of kicks, while they ought to be without a chance. Yet interpretation of success rates is not straightforward. Comparing success rates among goalkeepers to uncover underlying goalkeeping skill is thwarted because success also reflects the quality of a penalty taker's kick. Consequently, experimental researchers typically control for the quality of penalty kick attempts (e.g., by using virtual kicks presented with video; Savelsbergh et al., 2002). Alternatively, researchers have based the measure for goalkeeping performance on criteria other than success rate only. For example, Dicks et al. (2010a) developed a 5-point-scale: 0 points were assigned if the goalkeeper made a movement to the wrong goal side; 1 point was assigned if the goalkeeper did not move; two points were assigned if the goalkeeper moved to the right side, but did not dive and contact the ball; three points were assigned for a dive to the right side, but no contact; four points were assigned for a dive and contact, but no save; and five points were only warranted if a goalkeeper successfully saved the kick (no matter how).

However, even this measure is not without its flaws. Imagine two goalkeepers, A and B: goalkeeper A judges the side correctly but dives too late to reach the ball—for instance, because A waited until the penalty taker's intention became clear. By contrast, goalkeeper B dives to the wrong side but would have reached the ball in time if he jumped to the correct side. Based on success rate, performance is equally poor. Dicks et al. (2010a), however, would assign four points to A and zero points to B. Clearly, their measure prioritizes spatial decisions over temporal decisions. In fact, this is exemplary for most studies on goalkeeping in penalty kicks (van der Kamp et al., 2018). The point is that temporal decision making should also be taken into account, not on its own but together with the spatial aspects of decision making. There are only a few studies addressing how goalkeepers do and/or should time their dive to increase their success rate. In this respect, Kuhn (1988) compared goalkeepers who initiated their jump before or after the penalty taker contacts the ball and—based on observations of a small sample of high-skilled goalkeeper—concluded that goalkeepers who move late have a higher success rate. In reference to that study, Savelsbergh et al. (2010) argued that goalkeepers can take much of the penalty taker's actions into account and commit to one side rather late during the run-up and thus prioritize choosing the correct side. Alternatively, they can commit early and chose the side of the goal to defend before or during the run-up, merely guessing or relying on knowledge about the penalty taker's preferred side and thus prioritize deciding in time to reach a ball. In an experimental study, Savelsbergh et al. (2005) confirmed that more successful high-skilled goalkeepers waited longer before deciding about goal side than less successful counterparts. Further to this point, Furley et al. (2017) analyzed performance of goalkeepers at FIFA World Cups and UEFA European Championships. They also found that high-skilled goalkeepers usually waited long before initiating their jump. However, it also turned out that sometimes goalkeepers were “waiting too long.” With ball flight times sometimes being 500 ms or less (Franks and Harvey, 1997), systematically diving after the ball is contacted, that is, until the correct goal side to defend has become completely evident, is not always wise. Accordingly, Dicks et al. (2010b) reported that less agile goalkeepers have to commit themselves earlier to one side, simply because they require more time to dive to the ball (see also Zheng et al., 2021). In general, a goalkeeper's adequate temporal decision making reflects the interplay between the goalkeeper (i.e., time required to dive and block the ball) and the penalty taker (i.e., time made available to dive and block the ball) (Furley et al., 2017; van der Kamp et al., 2018). And this would be equally true for temporal decision making of the penalty taker (van der Kamp, 2011).

Penalty takers and goalkeepers make spatial and temporal decisions about goal side. Goalkeepers must (strategically) decide to dive early or wait longer for committing to one side or remaining in the middle of the goal (Savelsbergh et al., 2010), while penalty takers must decide prior to the run-up where to kick (i.e., keeper-independent strategy) or wait for the goalkeeper to commit to a side to subsequently kick to the other side of the goal (i.e., keeper-dependent strategy; van der Kamp, 2006; Noël and van Der Kamp, 2012; see also Kuhn, 1988). Theoretically, if penalty takers employ the keeper-independent strategy and do not change kick direction (and probably attempt to maximize accuracy and power of the kick), then goalkeepers may jump relatively early to increase the chance they are in time to block the ball in case they choose the correct goal side. They then would prioritize a higher likelihood of being in time to stop the ball over a higher likelihood of committing to the correct side. However, in doing so, it must be kept in mind that a well-placed (cf. Azar and Bar-Eli, 2011) and powerful kick is usually out of a goalkeeper's reach, also when they commit relatively early. By contrast, for penalty takers who employ a keeper-dependent strategy, it stands to reason that goalkeepers may wait longer before committing to one side, increasing the likelihood that they commit to the correct side. That is, research shows that the keeper-dependent strategy comes with an increased risk for the penalty taker of running out of time while deciding what side to kick in the case that goalkeepers initiate their movement relatively late. This may hamper the production of an accurate and powerful kick (van der Kamp, 2006, 2011; Noël and van Der Kamp, 2012).

In the current study, we examined the temporal interplay of goalkeepers and penalty takers. To this end, we analyzed the penalty taking (i.e., distinguishing between keeper-dependent and keeper-independent strategies) and goalkeeping strategies (i.e., distinguishing between diving early and late) in penalty shoot-outs at FIFA World Cups and UEFA European Championships, and we determined the success rates associated with these strategies (Noël et al., 2015). In doing so, we intended to assess the combined influence of goalkeepers' and penalty takers' strategies on scoring probabilities to scrutinize to what degree this interplay has to be considered when advising goalkeepers and penalty takers how to approach a penalty kick.

## Method

### Data

The data sample consisted of penalty kicks from all penalty shoot-outs at FIFA World Cups and UEFA European Championships between 1984 and 2016, totaling 41 penalty shoot-outs and 395 penalty kicks. In 1997, a relevant change in the FIFA penalty kick rules was introduced. The goalkeeper was no longer forced to stand still on the goal line. This amendment of the rules was actually a perpetuation of an existing practice, in which goalkeepers moved (sideward) on the goal line. As such, it is unlikely to have fundamentally affected the dynamics of the interplay between goalkeeper and penalty taker.

The FIFA World Cups and UEFA European Championships are two of the most important international tournaments, which ascertained that players in the penalty shoot-outs were highly engaged and motivated. The players were among the best in the world or continent, with their teams reaching the knockout phase in the tournament (i.e., matches that are decided on penalty kicks after a draw). Of these penalty kicks, 289 attempts (73.2%) were successfully converted, and the remaining 106 (26.8%) were either saved by the goalkeeper or missed the goal. Footage of the whole penalty shoot-outs was obtained from YouTube.com, private collections of TV broadcasts, and various other internet sources after verifying matches with penalty shoot-outs from the websites of FIFA and UEFA. For the majority of penalty kicks, the footage was directly from behind either the goal or the penalty taker. Camera perspective and quality of footage varied but always allowed reliable observation of the goalkeeper's movements (i.e., the onset of the final jump to one side relative to the moment the kicker contacts the ball, and dive direction) and the penalty taker's actions (i.e., fluency of the run-up, attention for the goalkeeper, kicking technique, and kick direction) as well as the outcome of the penalty kick (i.e., score, save, or miss).

### Analysis

To identify the penalty kick strategy, we used the three-predictor logistic regression model previously validated by Noël et al. (2015). In that study, skilled players were instructed to take penalty kicks adopting either a keeper-independent or keeper-dependent strategy. Afterwards, observers rated the video recordings of the penalty kicks to identify the factors that distinguished the two strategies. This showed that run-up fluency, a penalty taker's attention for the goalkeeper, and kicking technique reliably categorize a penalty kick as keeper-dependent or keeper-independent. The current study adopted this model. That is, two soccer coaches (both male, 37 and 36 years old) with 10 years of coaching experience on an intermediate amateur level independently rated the penalty takers' run-up fluency (i.e., stagnant–fluent) and attention to the goalkeeper (i.e., attention–no attention) by marking a location on continuous 11-point Likert scales and identified the kicking technique (i.e., instep, inside, or outside of the foot) of all 395 penalty kicks. The video footage was shown in random order to the two soccer coaches on a 15-inch monitor. Inter-rater reliability was high for both run-up fluency and attention (*r*'s > 0.85), and there were no disagreements with respect to kicking technique. Subsequently, the mean Likert scores of the two raters were used and submitted to the three-predictor logistic regression model that classifies each penalty kick as either keeper-independent or keeper-dependent (cf. Noël et al., 2015). This returned for each penalty kick the most likely penalty kick strategy employed by the penalty takers.

Next, QuickTime Player was used by one of the coaches to determine the moment of the goalkeeper's dive. For each kick, the frame at which the goalkeeper started the dive to the left or right and the frame of football contact by the penalty taker were identified. The soccer coach was not specifically instructed how to identify the goalkeepers' onset of movement, but it was emphasized that prior, preparatory, or deceptive movements before the final movement to one side had to be neglected. The first author of the current study also determined the moment of the goalkeeper's dive and the moment of football contact for a randomly chosen 10% of the penalty kicks to assess reliability of the analysis, which again was high for both variables (*r*'s > 0.85). The number of frames between football contact and movement onset was counted and multiplied by 40 ms (i.e., the duration of a single frame) to get the moment of initiation of the dive relative to the football contact. Goalkeepers who moved at or later than 160 ms before football contact were defined as “late responders” [i.e., they probably waited until they could identify that the penalty taker had placed the non-kicking leg next to the ball, which is considered the earliest reliable cue informing about the side to which the ball is kicked; see Savelsbergh et al. (2002), Lees and Owens (2011), and Diaz et al. (2012)], while goalkeepers who moved 200 ms before football contact or earlier were regarded as “early responders.” Late responders behave as if prioritizing side over time, whereas early responders may prioritize time over side. In case a goalkeeper remained in the middle of the goal, this was classified as a late responder (<3% of the penalty kicks). We run an additional analysis without these penalty kicks. This did not significantly affect the pattern of results.

## Results

With the use of the three-predictor logistic regression model and inputting the rating for attention to the goalkeeper, run-up fluency and kicking technique for each penalty kick classified 72.66% of the penalty kicks as a keeper-independent strategy and 27.34% as a keeper-dependent strategy. Goalkeepers responded early (i.e., 200 ms or longer before football contact) in 33.92% of the penalty kicks and were late responders (i.e., starting 160 ms or later before football contact) in 66.08% of the kicks. The success rate of penalty takers seemed similar for penalty kicks in which they employed keeper-independent (72.02%) and keeper-dependent strategies (74.65%). By contrast, the goalkeepers appeared more successful while moving early than late. That is, the success rate of the penalty takers was 68.72 vs. 76.30%. Further to this point, Table 1 shows the success rates of penalty takers (i.e., score/no score) for the four combinations of penalty kick and goalkeeping strategy. A χ^2^ test on the number of penalties scored showed that the combination of both strategies had an impact on scoring, $\chi_{\left(1\right)}^{2}$ = 2.259; *p* = 0.039, Cramer's *V* = 0.121. The probability of a score was high (81.81%) when the goalkeepers moved early and at the same time the penalty takers were employing a keeper-dependent strategy, but also when the goalkeepers moved late while the penalty takers employed a keeper-independent strategy (79.69%). In contrast, penalty takers scored less often when goalkeepers moved early and they were employing a keeper-independent strategy (60.00%), or when goalkeepers moved late while penalty takers were employing a keeper-dependent strategy (63.63%).

**Table 1:** Scoring rates and number of converted penalties (in brackets) as a function of penalty kick strategy (keeper-dependent vs. keeper-independent) and goalkeeping strategy (early vs. late movement).

	**Penalty kick strategy**	
	**Keeper-dependent**	**Keeper-independent**
Goalkeepers moving early	81.81% (36)	60.00% (54)
Goalkeepers moving late	63.63% (42)	79.69% (157)

Furthermore, a second χ^2^ test on distributions of penalty kick strategies showed that goalkeepers' movement onset did not depend on penalty kick strategies. That is, the goalkeepers did not adapt the timing of the start of the dive to the penalty takers strategy [$\chi_{\left(1\right)}^{2}$ = 3.08; *p* = 0.079; Table 2]. However, it seems noteworthy that in cases that the penalty takers employed a keeper-independent strategy, goalkeepers behaved seemingly more adaptively (i.e., jumping to one side rather early) in only 31.4% of these penalties. In the case that the penalty takers employed a keeper-dependent strategy, goalkeepers behaved seemingly more adaptively (i.e., leaving the penalty taker unclear about their intentions by committing to one side late) in 59.3% of these cases.

**Table 2:** Distributions of goalkeeper strategies (moving early or late) and penalty kick strategy (keeper-dependent, keeper-independent).

	**Penalty kick strategy**	
	**Keeper-dependent**	**Keeper-independent**
Goalkeepers moving early	44	90
Goalkeepers moving late	64	197

## Discussion

The current paper scrutinized the temporal interplay of goalkeepers and penalty takers' decision making. Goalkeepers must consider the penalty taker's run-up for deciding when to initiate their jump to the ball, while penalty takers decide where to kick the ball either prior to the run-up or wait until the goalkeeper has committed to one side. We analyzed video footage of actual penalty kicks during FIFA World Cups and UEFA European Championships.

The observational analyses showed that the professional goalkeepers were more inclined to move late (i.e., after the non-kicking leg is placed next to the ball, ~160 ms before ball contact) than early (i.e., 66 vs. 34%, respectively). At first sight, this accords with observations from experimental studies, which show that more successful goalkeepers wait relatively long before they commit themselves to one side (e.g., Savelsbergh et al., 2005). It is presumed that more successful goalkeepers wait longer to decide for a goal side, because this allows them to access more reliable information from the penalty taker's kicking actions to anticipate the penalty takers' intentions. That is, the orientation of the non-kicking foot informs in over 80% about kick direction, while subsequent movement of the kicking leg provides even more reliable information (Diaz et al., 2012). Possibly, high-skilled goalkeepers can do this because they are more agile and, thus, can cover more space in the short time available (Dicks et al., 2010a; Zheng et al., 2021). By contrast, when jumping early, spatial decisions can only be based on less or unreliable information, such as the angle of the run-up (cf. Loffing and Hagemann, 2014), and makes goalkeepers also more susceptible for penalty takers' attempts to deceive goalkeepers (Dicks et al., 2010a). Alternatively, they can use knowledge about the penalty taker's preferred kicking side (Navia et al., 2013). Based on this work, the typical recommendation is that goalkeepers should wait as long as possible, because it provides more reliable information and also because waiting long makes penalty takers employing a keeper-dependent strategy more apt to produce a weak and inaccurate shot (van der Kamp, 2006, 2011). However, considering that penalty takers employ a keeper-independent strategy in the majority of penalty kicks, it is less surprising that goalkeepers were more successful jumping early than rather late. Remaining longer in the middle of the goal probably does not negatively affect the penalty taker who has adopted a keeper-independent strategy, but it does interfere with the goalkeepers' own ambitions of reaching the ball in time. By waiting longer for committing to one side of the goal, they have less time to stop the ball, and even if they would jump more often to the right side (because they are able to consider more reliable information, but see Zheng et al., 2021), then this would not allow them to save more penalty kicks. In other words, in their decision making, goalkeepers tend to prioritize choosing the right goal side over jumping early enough, undervaluing the importance of being in time to be able to block the ball (see also van der Kamp et al., 2018). We speculate that goalkeepers may behave in this way because they intend to show that—in any case—they are capable of choosing the correct side (i.e., not unlike the observation that goalkeepers always dive, though remaining in the center of the goal from time to time would increase their chances; Bar-Eli et al., 2007). This strategy seems especially detrimental given the actual distribution of penalty kick strategies.

The video analyses showed that penalty takers employed the keeper-dependent strategy (i.e., waiting until goalkeepers initiate their dives) in only 27% of the kicks, while they adopted a keeper-independent strategy in 73% of the penalty kicks. This confirms the previously reported prevalence in the use of the keeper-independent strategies among professional players (Noël et al., 2015) and is also in agreement with experimental studies showing that the use of keeper-independent strategy is less risky and more effective (van der Kamp, 2006; Wood and Wilson, 2010; Noël and van Der Kamp, 2012). By choosing goal side before the run-up, the keeper-independent strategy minimizes the risk for running out of time when adjusting kick direction to the goalkeepers' actions as per keeper-dependent strategy (van der Kamp, 2006). Additionally, gaze during the keeper-independent strategy is more often directed at the target and the ball (rather than the goalkeeper), allowing for a more accurate kick (Hüttermann et al., 2014). Consequently, a previous work has strongly advised the use of a keeper-independent strategy. However, the current findings indicate that it is probably wise to interchange the two penalty kick strategies to a certain extent. In fact, success rates of employing keeper-independent and keeper-dependent strategy were quite similar (75 vs. 72%) but depended heavily on the goalkeeper's behavior. Considering that goalkeepers commit to one side of the goal relatively late in two thirds of penalty kicks, it remains wise to employ keeper-independent strategy more often given that in these cases keeper-independent strategy seems to be more effective. However, a keeper-independent strategy appears less effective in the case goalkeepers move early. Accordingly, leaving goalkeepers uncertain about the penalty kick strategy they can expect seems of great importance. Otherwise, goalkeepers can adapt their strategy to preferences of individual penalty takers (i.e., presuming that such information is available to the goalkeeper). As long as a penalty taker is able to place the ball accurately and with power, it would not affect his chances, but in cases a ball is less well placed (as is not unlikely in stressful situations), it would certainly increase the goalkeepers' chances of reaching a ball in time.

This being said, the overrepresentation of the keeper-independent strategy in the most competitive tournaments might also be due to many non-specialists being assigned to take a penalty kick during penalty shoot-outs. A keeper-independent strategy increases perceived control of the situation, because it allows planning the details of the kick beforehand rather than being dependent upon unpredictable behaviors of the goalkeeper. Perception of control has been shown to be critical for maintaining performance in stressful situations, also in professional soccer (e.g., Jordet et al., 2006). Notwithstanding this, the current study is the first to suggest that using a keeper-dependent strategy may (under certain circumstances) be more favorable than using keeper-independent strategy, but the findings also underline that in the history of FIFA World Cups and UEFA European Championships both strategies were associated with a similar chance to score (cf. Noël et al., 2015) and, importantly, that alternating strategies seems important for continued success of penalty takers (and, similarly, for goalkeepers). Insufficient alternation allows the opponent to readily adapt to the preferred strategy.

The observed temporal interplay between players during penalty kicks appears consistent with the claim of van der Kamp et al. (2018) in that successfully performing an interceptive action like saving a penalty kick depends not only on spatial decision making (e.g., choosing the left or right side of the goal) but should be carefully balanced with the temporal aspects (e.g., deciding when to dive). Given the lack of research on these temporal aspects, we cannot be sure if currently professional players' decisions are indeed constrained by deliberations or strategies regarding timing, as typically done with respect to choosing goal side. Anecdotally, the German goalkeeper Jens Lehman used a cribbage during the penalty shoot-out in the 2006 World Cup quarterfinal against Argentina. The cribbage not only mentioned the Argentinian players' preferred kicking side but also informed Lehman about the desired temporal strategy, such as “wait long” (“*lange warten*”). Whether this is representative for penalty takers' and goalkeepers' preparations for a penalty kick remains for further research. In any case, it seems wise to have this information available.

In sum, the current analysis adds to previous findings from sport psychology and sport science. This leads to subtle but important reconsiderations of existing recommendations, for maximizing performance in the penalty kick for both goalkeepers and penalty takers. Specifically, goalkeepers may increase their success rate by more often starting their jump early than they currently do. Penalty takers are advised to alternate the use of keeper-dependent and keeper-independent strategies instead of relying too strongly on the keeper-independent strategy, as was previously recommended.

## Data Availability Statement

The raw data supporting the conclusions of this article will be made available by the authors, without undue reservation.

## Author Contributions

BN performed the statistical analysis and wrote the first draft of the manuscript. All authors contributed to the conception and design of the study. All authors wrote sections of the manuscript, contributed to manuscript, and read/approved the submitted version.

## Conflict of Interest

The authors declare that the research was conducted in the absence of any commercial or financial relationships that could be construed as a potential conflict of interest.
